# Factors predisposing to claims and compensations for patient injuries following total hip and knee arthroplasty

**DOI:** 10.3109/17453674.2012.672089

**Published:** 2012-04-24

**Authors:** Jutta Järvelin, Unto Häkkinen, Gunnar Rosenqvist, Ville Remes

**Affiliations:** ^1^Center for Health and Social Economics CHESS, National Institute for Health and Welfare; ^2^Department of Finance and Statistics, Hanken School of Economics; ^3^Department of Orthopedics and Traumatology, Peijas Hospital, Helsinki University Central Hospital, Helsinki, Finland

## Abstract

**Background and purpose:**

Factors associated with malpractice claims are poorly understood. Knowledge of these factors could help to improve patient safety. We investigated whether patient characteristics and hospital volume affect claims and compensations following total hip arthroplasty (THA) and knee arthroplasty (TKA) in a no-fault scheme.

**Methods:**

A retrospective registry-based study was done on 16,646 THAs and 17,535 TKAs performed in Finland from 1998 through 2003. First, the association between patient characteristics—e.g., age, sex, comorbidity, prosthesis type—and annual hospital volume with filing of a claim was analyzed by logistic regression. Then, multinomial logistic regression was applied to analyze the association between these same factors and receipt of compensation.

**Results:**

For THA and TKA, patients over 65 years of age were less likely to file a claim than patients under 65 (OR = 0.57, 95% CI: 0.46–0.72 and OR = 0.65, CI: 0.53–0.80, respectively), while patients with increased comorbidity were more likely to file a claim (OR = 1.17, CI: 1.04–1.31 and OR = 1.14, CI: 1.03-1.26, respectively). Following THA, male sex and cemented prosthesis reduced the odds of a claim (OR = 0.74, CI: 0.60–0.91 and OR = 0.77, CI: 0.60–0.99, respectively) and volume of between 200 and 300 operations increased the odds of a claim (OR = 1.29, CI: 1.01–1.64). Following TKA, a volume of over 300 operations reduced the probability of compensation for certain injury types (RRR = 0.24, CI: 0.08–0.72).

**Interpretation:**

Centralization of TKA to hospitals with higher volume may reduce the rate of compensable patient injuries. Furthermore, more attention should be paid to equal opportunities for patients to file a claim and obtain compensation.

Despite the high frequency of adverse events in healthcare, only a small proportion of patients who have experienced such an event file a claim for damages (Localio et al. 1991, Studdert et al. 2000, Bismark et al. 2006). The reasons for filing or not filing a claim are not fully understood. Moreover, studies on this issue have so far been done mainly in countries where malpractice claims are handled by the tort system, and they have typically dealt with health services at an aggregate level without much regard to individual medical or surgical procedures (Localio et al. 1991, Studdert et al. 2000). Information of this type, however, would be useful in anticipating adverse events and in targeting patient safety measures accurately to specific procedures.

The aim of this study was to determine whether patient characteristics, prosthesis type, and hospital volume affect filing a claim and receiving compensation in a no-fault (also called no-blame) insurance scheme following total primary hip arthroplasty (THA) or total primary knee arthroplasty (TKA). These procedures were chosen because they are among the most common types of surgical procedures for which patients file a claim.

According to previous studies, male patients and elderly patients are less likely to claim for damages (Studdert et al. 2000, Pukk et al. 2003, Bismark et al. 2006, Järvelin et al. 2009), while patients with increased comorbidity are more likely to do so (Järvelin et al. 2009). We hypothesized that this would also be the case with regard to THA and TKA. Furthermore, we assumed that cemented prosthesis and hospital volume have a negative association with claims because cemented prostheses have involved less short-term complications than uncemented prostheses (Morshed et al. 2007, Hailer et al. 2010) and because the quality of arthroplasty surgery has been reported to be better at high-volume hospitals (e.g., Shervin et al. 2007).

## Patients and methods

Information on patients who had undergone primary THA or TKA at a public hospital (including some large health centers) in Finland between 1998 and 2003 was retrieved from the Hospital Discharge Register using mostly the same criteria that were used in a previous study on the cost-effectiveness of arthroplasty surgery (Mäkelä et al. 2011). Cases were included if the diagnosis codes indicated primary osteoarthritis of the hip (ICD-10 codes M16.0 or M16.1) together with a procedure code of THA (NFB30–NFB60 or NFB99, according to the Nordic Medico-Statistical Committee's classification). With regard to TKA, the corresponding diagnosis code was M17.0 or M17.1, together with a procedure code of TKA (NGB10–NGB99).

Next, we excluded patients where (1) medical history as observable from the Hospital Discharge Register and the Social Insurance Institution's registers contained codes representing secondary osteoarthritis (3,614 patients) (precise codes are available from the authors upon request); (2) arthroplasty took place simultaneously on both hip and knee (37 patients); (3) prosthesis type was unknown (1,840 patients); (4) records in the Hospital Discharge Register indicated that their place of residence was the Åland Islands (this exclusion is typical of Finnish studies, as the Åland Islands are an autonomous region and also because a number of patients undergo surgery in Sweden, with precise figures being unavailable) (177 patients). Furthermore, if patients had had 2 primary THAs (TKAs) in the study period but at different points in time (5,238 patients), the latter operation was excluded (as including both would not have ensured independent observations). Thus, the final dataset comprised 16,646 THA patients and 17,535 TKA patients.

### The patient injury insurance scheme in Finland

In Finland, healthcare-related claims for damages, amounting to some 7,000 to 8,000 cases per year, are handled by the Patient Insurance Center (Patient Insurance Center 2011). In making its decisions, the center uses various sources of information such as patient records, radiographs, statements by external medical experts, and most notably the Patient Injury Act of 1987, amended in 1999.

The Patient Injury Act describes 7 criteria in which compensation would be considered justified. The first and most commonly applied criterion is a treatment injury, which is defined as compensable if the patient's care did not measure up to the standard of an experienced health professional, and was therefore preventable. The remaining 6 criteria include infection injury, accidental injury such as an accident during ambulance transportation, defective equipment, damage to healthcare facilities, deficiencies in the delivery of a pharmaceutical (except for unexpected side effects of pharmaceuticals, for which a separate compensation scheme exists), and unreasonable injury. None of these criteria require proof of negligence.

The legislative amendment to the Patient Injury Act in 1999 mainly concerned the compensation of injuries involving an infection. An infection had been compensable before the amendment on the basis of criteria similar to those for a treatment injury, i.e. on the basis of preventability. Preventability, however, proved to be an unsuitable measure in the case of an infection (Palonen et al. 2005). Consequently, its definition was revised and the main criterion for an infection injury became tolerability. This meant that for an infection to be compensable, it had to be unexpected as assessed from the patient's past and current medical problems, the treatment given, and other factors defined by the Patient Injury Act.

### Linking claims with THA and TKA admissions

We obtained data on claims for injuries that occurred between 1998 and 2003 from the register of the Patient Insurance Center. These data included the patient's social security number, the date of injury, hospital, diagnosis, and procedure codes; these were then used to link claims to THA or TKA over several stages (a detailed description of the linkages is available from the authors upon request). Subsequently, we discovered 408 THAs (2.5%) and 437 TKAs (2.5%) that had led to a claim. Of these, 198 (49%) were for successful THA-related claims and 182 (42%) were for successful TKA-related claims. Furthermore, for successful claims following THA, 148 (75%) were for treatment injuries and the remainder for infection injuries, while regarding TKA, 102 (56%) were for treatment injuries and the remainder for infection injuries (Table 1).

**Table 1.** Descriptive statistics on THA and TKA patients operated on in the public sector in Finland from 1998 through 2003. Values are mean (SD) or n (%)

		All patients		Patients having filed a claim	
		THA **^a^**	TKA **^b^**	THA **^a^**	TKA **^b^**
	Description	(n =16,646)	(n = 17,535)	(n = 408)	(n = 437)
*Explanatory variable*					
Age over 65	Aged over 65 = 1, aged 65 or less = 0	10,680 (64%)	13,260 (76%)	206 (50%)	296 (68%)
Male	Male = 1, female = 0	7,144 (43%)	4,784 (27%)	159 (39%)	108 (25%)
Charlson index	A value from 0 to 11 (in this study population) depending on the number and/or severity of comorbidities	0.38 (0.79)	0.44 (0.85)	0.43 (0.77)	0.53 (0.97)
Cemented	Both components of the prosthesis cemented = 1, otherwise 0	8,091 (49%)	16,314 (93%)	160 (39%)	405 (93%)
Hybrid	Either component of the prosthesis cemented and the other not = 1, otherwise 0	2,710 (16%)	808 (5%)	70 (17%)	26 (6%)
Previous THA (TKA)	Contralateral primary THA (TKA) before current THA (TKA) = 1, otherwise 0	1,870 (11%)	1,806 (10%)	48 (12%)	35 (8%)
THA (TKA) before change in law	THA (TKA) before the amendment to the Patient Injury Act = 1, otherwise 0	3,662 (22%)	3,561 (20%)	87 (21%)	82 (19%)
Volume < 200	THA (TKA) performed at a hospital with less than 200 of these operations per year = 1, otherwise 0; reference category for volume 200–300 and volume > 300	12,715 (76%)	12,902 (74%)	297 (73%)	324 (74%)
Volume 200–300	THA (TKA) performed at a hospital with more than 200 but less than 300 of these operations per year = 1, otherwise 0	3,042 (18%)	2,587 (15%)	90 (22%)	67 (15%)
Volume > 300	THA (TKA) performed at a hospital with more than 300 of these operations per year = 1, otherwise 0	889 (5%)	2,046 (12%)	21 (5%)	46 (11%)
*Explained variable*					
Claim	Filed a claim = 1, otherwise 0	408 (2.5%)	437 (2.5%)	–	–
Outcome of claim					
Treatment injury	Obtained compensation for a treatment injury = 1, otherwise 0	–	–	148 (36%)	102 (23%)
Infection injury	Obtained compensation for an infection injury = 1, otherwise 0	–	–	50 (12%)	80 (18%)
No compensation	Did not obtain any compensation = 1, otherwise 0			210 (51%)	255 (58%)

**^a ^**THA: total hip arthroplasty.

**^b ^**TKA: total knee arthroplasty.

### Study variables

The explanatory variables comprised age over 65 years, sex, comorbidities, prosthesis type (obtainable from the Finnish Arthroplasty Register and depicted by 2 variables, cemented and hybrid), whether the patient had had a primary contralateral joint replacement previously, whether the patient's arthroplasty took place before the amendment to the Patient Injury Act, and hospital volume (Table 1).

Comorbidities were measured with the Charlson comorbidity index (Charlson et al. 1987). In this study, it included patient's comorbidities recorded in the Hospital Discharge Register from 1987 up until the arthroplasty admission, but not those recorded at the admission. Comorbidities of the arthroplasty admission were not included in the index, to avoid including surgical complications.

For variables depicting hospital volume, we divided the hospitals into 3 groups: hospitals with less than 200 arthroplasties per year (on average, 45 hospitals during the study period); those with more than 200 arthroplasties but less than 300 per year (on average, 5 hospitals); and those with more than 300 arthroplasties per year (on average, 3 hospitals). Consequently, 3 variables indicated in which of the 3 hospital groups a patient's operation took place; the reference category was an annual volume of less than 200 operations. It is noteworthy that the number of arthroplasties comprised all primary THAs and TKAs irrespective of diagnosis, and was calculated separately for hip and knee.

The explained variable was a dichotomous variable: 1 if the patient had filed a claim and 0 if the patient had not filed a claim. In a subsequent statistical analysis, the explained variable comprised 3 categories describing the outcome of the patient's claim: no compensation (denoted by 0, reference category), compensation for a treatment injury (1), and compensation for an infection injury (2). This categorization was adopted because the decision regarding compensability is an assessment of whether one of the 7 criteria of compensation in the Patient Injury Act is met rather than an assessment of whether a claim is compensable or not.

### Statistics

We conducted the statistical analyses in 2 stages (Figure). In the first stage, logistic regression analysis was used to analyze the effect of patient characteristics, prosthesis type, and hospital volume on filing of a claim. In the second stage, data were restricted to the subset of patients who had filed a claim, to which we applied multinomial logistic regression analysis. This analysis yielded a relative risk ratio (RRR, a ratio of 2 relative risks) for each explanatory variable as well as for the 2 comparisons: treatment injury category against no compensation and infection injury against no compensation. For instance, the RRR for individuals older than 65 while comparing treatment injury against no compensation was the ratio of the 2 relative risks: (1) the probability of treatment injury divided by the probability of no compensation if the patient is over 65, and (2) the probability of treatment injury divided by the probability of no compensation if the patient is under 65. In the case of the Charlson index—a count variable—the RRR similarly measured the ratio of relative risks when the index increased by 1.

**Figure:**
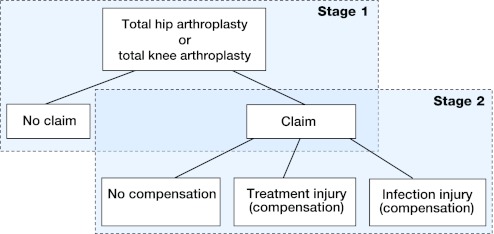
Stages in the statistical analysis; the first stage used logistic regression analysis and the second multinomial logistic regression analysis.

We carried out the statistical analyses using StataSE 10 software and regarded a p-value of less than 0.05 as statistically significant.

### Ethics

The study was approved on April 18, 2002 by the Ethics Committee of STAKES, a predecessor of the Finnish National Institute for Health and Welfare.

## Results

In the logistic regression analyses regarding THA, patients who were over 65 years of age, men, and patients with a cemented prosthesis were less likely to file a claim and patients who had been operated on at a hospital with an annual volume of between 200 and 300 operations were more likely to file a claim. Likewise, regarding TKA, those patients who were over 65 years of age were less likely to file a claim. Furthermore, regarding both THA and TKA, a 1-unit increase in the Charlson index increased the odds of a claim by 17% and 14%, respectively (Table 2).

**Table 2.** Results of the first stage using logistic regression analysis^a^

		THA **^b^**			TKA **^c^**	
		(n = 16,646)			(n = 17,535)	
Variable	OR **^d^**	95% CI	p-value	OR **^d^**	95% CI	p-value
Age over 65	0.57	0.46–0.72	< 0.001	0.65	0.53–0.80	< 0.001
Male	0.74	0.60–0.91	0.004	0.82	0.66–1.03	0.09
Charlson index	1.17	1.04–1.31	0.008	1.14	1.03–1.26	0.01
Cemented	0.77	0.60–0.99	0.04	1.70	0.75–3.85	0.2
Hybrid	0.92	0.69–1.23	0.6	2.23	0.91–5.47	0.08
Previous THA (TKA)	1.15	0.85–1.57	0.4	0.80	0.56–1.13	0.2
THA (TKA) before change in law	0.96	0.75–1.22	0.7	0.92	0.72–1.18	0.5
Volume 200–300	1.29	1.01–1.64	0.04	1.02	0.78–1.34	0.9
Volume > 300	1.02	0.65–1.62	0.9	0.85	0.62–1.17	0.3

**^a^** Explained variable 1 if the patient filed a claim, otherwise 0.

**^b^** THA: total hip arthroplasty.

**^c^** TKA: total knee arthroplasty.

**^d^** OR: odds ratio.

In the multinomial regression analysis, none of the study variables were statistically significant with regard to THA whereas several variables were statistically significant with regard to TKA. A hospital volume of over 300 operations compared to a volume of less than 200 operations was associated with a reduced relative risk of compensated treatment injury relative to no compensation. Furthermore, the relative risk of a compensated treatment injury was lower for patients over 65 years of age compared to patients under 65, while the relative risk of a compensated infection injury was higher for men than for women. In addition, the relative risk of a compensated infection injury was higher for patients with a previous contralateral operation than for those without such an operation (Table 3).

**Table 3.** Results of the second stage using multinomial logistic regression analysis^a^

		THA **^b^**			TKA **^c^**	
*Outcome of claim*		(n = 408)			(n = 437)	
Variable	RRR **^d^**	95% CI	p-value	RRR **^d^**	95% CI	p-value
* Treatment injury*						
Age over 65	0.64	0.39–1.05	0.08	0.57	0.34–0.94	0.03
Male	1.08	0.69–1.69	0.7	0.74	0.41–1.34	0.3
Charlson index	1.10	0.84–1.45	0.5	0.99	0.78–1.27	1.0
Cemented	1.25	0.73–2.15	0.4	0.37	0.05–2.79	0.3
Hybrid	0.93	0.49–1.76	0.8	0.12	0.01–1.26	0.08
Previous THA (TKA)	1.58	0.82–3.03	0.2	2.46	1.00–6.07	0.05
THA (TKA) before change in law	1.05	0.62–1.80	0.9	1.39	0.76–2.54	0.3
Volume 200–300	0.97	0.58–1.63	0.9	0.64	0.32–1.29	0.2
Volume > 300	1.03	0.39–2.69	1.0	0.24	0.08–0.72	0.01
* Infection injury*						
Age over 65	1.03	0.49–2.18	0.9	0.96	0.54–1.73	0.9
Male	1.83	0.96–3.49	0.07	2.07	1.18–3.64	0.01
Charlson index	0.86	0.55–1.34	0.5	0.99	0.75–1.30	0.9
Cemented	2.00	0.89–4.50	0.09	0.26	0.03–1.97	0.2
Hybrid	1.13	0.43–3.02	0.8	0.39	0.04–3.60	0.4
Previous THA (TKA)	1.23	0.46–3.27	0.7	4.02	1.70–9.55	0.002
THA (TKA) before change in law	2.02	1.00–4.09	0.05	1.86	0.98–3.53	0.06
Volume 200–300	0.95	0.44–2.05	0.9	1.49	0.74–2.99	0.3
Volume > 300	0.86	0.18–4.14	0.8	0.97	0.41–2.32	1.0

**^a^** Explained variable 0 if the patient did not obtain any compensation, 1 if the patient obtained compensation for a treatment injury, 2 if the patient obtained compensation for an infection injury; reference category = 0.

**^b^** THA: total hip arthroplasty.

**^c^** TKA: total knee arthroplasty.

**^d^** RRR: relative risk ratio.

Since the Helsinki University Central Hospital seemed to be an outlier with its notably high volume, we also conducted the analyses without this hospital. With this omission, for THA, a hospital volume of over 300 operations had an association with filing a claim (with an OR of 2.6, 95% CI; 1.4–4.7; p = 0.002). Furthermore, in the multinomial logistic regression analysis on TKA, the statistical significance of the volume variable over 300 operations weakened such that the RRR for treatment injury was 0.32 (p = 0.08).

The log likelihood ratio test and the Hosmer-Lemeshow test (not reported here) indicated a good model fit in all of the statistical analyses except for the multinomial logistic regression analysis with regard to THA. Here, based on the log likelihood ratio test, the model fit was poor.

## Discussion

We found that elderly patients were less inclined to file claims than younger patients. Various explanations have been proposed for this previously, ranging from elderly patients' lower economic loss from injuries to acceptance of poor outcomes at the end of one's life (Studdert et al. 2000, Bismark et al. 2006, Järvelin et al. 2009). In contrast, the reason for the larger odds of patients with increased comorbidity filing a claim could be that individuals who are more sick are generally less content with their medical care and—based on their more extensive use of health services—may have better knowledge of various ways of complaining than healthier people (Jackson et al. 2001, Xiao and Barber 2008, Rahmqvist and Bara 2010). Even so, comorbidity did not have a statistically significant effect on receiving compensation.

Some comment on comorbidity is necessary, though. The Charlson index did not perhaps cover all of the risk factors that are relevant in arthroplasty surgery; good examples are hyperglycemia (not just a history of diabetes) and obesity. But then again, the impact of patients' comorbidities on the outcome and occurrence of complications of arthroplasty surgery is ambiguous and may generally be small (Jones et al. 2007).

With regard to THA, filing of a claim was less likely with a cemented prosthesis. Its advantages, such as fewer early periprosthetic fractures and a minor tendency to result in leg length inequality, could have contributed to the result (Berry 1999, Ahmad et al. 2009). The result may also have been affected by the smaller variety of different stems, necks, and off-sets that were available for uncemented prostheses and by surgeons' lesser experience with this prosthesis type in the study period. However, long-term differences between various types of prosthesis probably did not influence the result, as patients must file their claim within 3 years of their injury.

In contrast to the logistic regression analysis, the results of the multinomial logistic regression analysis varied greatly between THA and TKA. Why these procedures that otherwise have so many features in common should produce such different results could have several explanations. First, the overall rate of claims is very small, which makes it difficult to identify associated factors by statistical means. Second, assessment of whether patient care measured up to professional standards and whether the injury was preventable is sometimes difficult even for experts. Thus, it is possible that the compensability of similar cases could be judged differently; this is despite measures that aim for standardization of compensation practices, such as the establishment of the Patient Injuries Board, which is an independent expert group that directs compensation settlements by releasing recommendations and statements.

One of the variables where the coefficient and its statistical significance differed clearly between THA and TKA was hospital volume. As is known from previous studies, hospital volume might have a smaller influence on the outcome of THA than surgeon volume, while the reverse might be true for other orthopedic procedures (Shervin et al. 2007). Moreover, with regard to THA, higher hospital volume has been found to be associated only with lower mortality and dislocation rate (Katz et al. 2001, Battaglia et al. 2006, Doro et al. 2006, Shervin et al. 2007), whereas with regard to TKA, hospital volume has been reported to be inversely related to a much wider range of adverse outcomes such as mortality, re-admission due to infection, pulmonary embolism, thrombophlebitis, pneumonia, anemia, and early revision (Norton et al. 1998, Kreder et al. 2003, Katz et al. 2004, Soohoo et al. 2006). Overall, the outcomes for TKA are not quite as good as for THA (Ethgen et al. 2004, Pulido et al. 2008, Bourne et al. 2010), though this difference has not been found in every study (Jones et al. 2001, Fehringer et al. 2010).

If hospital volume in general has a greater effect on adverse events in TKA than in THA, this could explain the statistically significant association between compensation and volume in the case of TKA but not THA. Why this association was apparent only for treatment injuries but not for infection injuries could possibly be explained by the lower rate of infections, by the assumption that hospital volume has a less important effect on infections, or by the assumption that infections are affected only by very large volumes—volumes that did not occur in this study. Irrespective of the underlying reasons, the result supports the view that TKAs should take place at hospitals with a higher volume of such procedures, in order to both reduce adverse events and to improve quality (Losina et al. 2009, Marlow et al. 2010).

In this study, the number of hospitals with high volume—that is, with more than 400 or 500 operations per year—was so small (2 hospitals, and even these did not perform over 400 operations every year) that it was not possible to estimate whether the rate of compensated claims would decrease linearly with increasing volume, or whether a certain volume would minimize the rate of compensated claims.

Omission of Helsinki University Central Hospital from the statistical analyses changed the results slightly, thus confirming its outlier position. Whether the influence of the hospital is derived directly from its higher volume or from its other characteristics, such as being a prominent university hospital, would necessitate further research. At the same time, smaller-sized hospitals appear to produce claims following THA less frequently, possibly because at these hospitals relationships between patients and healthcare personnel may be more congenial. Furthermore, the patient-safety culture, the activities of the patient ombudsman, and the ways in which adverse events are processed at a hospital may influence patients' decisions about whether or not to file a claim.

Based on our study, 2.5% of THA and TKA admissions lead to a claim, which is clearly a smaller rate than the overall complication rate from THA or TKA. Taken alone, short-term postoperative complications occur in 7.6% of THAs and 6.8% of TKAs (Fehringer et al. 2010). This is consistent with previous studies, according to which at most 3% of patients who have suffered an adverse event deserving compensation will file a claim (Localio et al. 1991, Studdert et al. 2000, Bismark et al. 2006).

At the same time, the 2.5% mentioned above indicates that a claim occurs much more frequently following THA and TKA than following hospitalizations in general. When hospitalizations are viewed together, a claim follows from only 0.1% to 0.2% of admissions (Localio et al. 1991, Studdert et al. 2000, Bismark et al. 2006, Pukk-Härenstam et al. 2008).

Regarding compensations for THA and TKA, the percentage of successful claims as a share of claims (at 49% and 42%, respectively) exceeds the comparable percentage for successful claims as a share of all claims, which in Finland is about 30% (Patient Insurance Center 2011). Furthermore, the distribution of successful claims between different injury types following THA and TKA differs from that of all successful claims in Finland. More than 90% of compensations for injuries in Finland are for treatment injuries, and less than 10% are for infection injuries, whereas in the case of THA, the percentages are 75% and 25%, respectively, and for TKA, 56% and 44%, respectively. Interestingly, the share of infection injuries following THA has been found to be even larger in Norway, at about 40% (Thomsen and Walloe 2010). This larger share may arise from a different incidence of infections or from different criteria for compensation.

Several factors that are known to affect the outcome of arthroplasty—such as surgical technique, rehabilitation after surgery, and the patient's socioeconomic status—were not included in the present study because these data were not available. Furthermore, the results regarding prosthesis types may be different in a similar study in the future because the design and survival of uncemented prostheses are improving continuously.
